# Utility of a ready-to-use PCR system for neuroendocrine tumor diagnosis

**DOI:** 10.1371/journal.pone.0218592

**Published:** 2019-06-27

**Authors:** Mark Kidd, Ignat A. Drozdov, Somer Matar, Nicole Gurunlian, Nicholas J. Ferranti, Anna Malczewska, Philip Bennett, Lisa Bodei, Irvin M. Modlin

**Affiliations:** 1 Wren Laboratories, Branford, Connecticut, United States of America; 2 Sarah Cannon Molecular Diagnostics, London, United Kingdom; 3 Medical University of Silesia, Katowice, Poland; 4 Memorial Sloan Kettering Cancer Centre, New York, New York, United States of America; 5 Yale University, School of Medicine, New Haven, Connecticut, United States of America; University of Helsinki, FINLAND

## Abstract

**Background:**

Multigene-based PCR tests are time-consuming and limiting aspects of the protocol include increased risk of operator-based variation. In addition, such protocols are complex to transfer and reproduce between laboratories.

**Aims:**

Evaluate the clinical utility of a pre-spotted PCR plate (PSP) for a novel multigene (*n* = 51) blood-based gene expression diagnostic assay for neuroendocrine tumors (NETs).

**Methods:**

A pilot study (*n* = 44; 8 controls and 36 NETs) was undertaken to compare C_Q_, normalized gene expression and algorithm-based output (NETest score). Gene expression was then evaluated between matched blood:tumor tissue samples (*n* = 7). Thereafter, two prospective sets (diagnostic: *n* = 167; clinical validation: *n* = 48, respectively) were evaluated for diagnostic and clinical utility value. Two independent molecular diagnostics facilities were used to assess assay reproducibility and inter-laboratory metrics. Samples were collected (per CLIA protocol) processed to mRNA and cDNA and then either run per standard assay (liquid primers) or on PSPs. Separately, matching plasma samples were analyzed for chromogranin A (CgA). Statistics included non-parametric testing, Pearson-concordance, Predictive Modeling and AUROC analyses.

**Results:**

In the pilot study (*n* = 44), C_Q_ values were highly concordant (r: 0.82, *p*<0.0001) and normalized gene expression data significantly related (*p*<0.0001) (Pearson-pairwise correlation). NETest values were not different (49.7±33 standard vs. 48.5±31.5 PSP) and the overall concordance in output 96%. Predictive modelling confirmed this concordance (F1 score = 0.95). Gene expression levels were highly correlated between blood and tumor tissue (R: 0.71–0.83). In the diagnostic cohort (*n* = 30 controls, *n* = 87 non-NET controls, *n* = 50 NET), NETest was significantly lower (*p*<0.0001) in controls (11±6.5) and non-NET controls (13±18) than NETs (61±31). The AUROCs were 0.93–0.97 and the diagnostic accuracy was 90–97.5%. As a diagnostic, the PSP-NETest was significantly better than CgA (accuracy: 56%, *p*<0.0001). For clinical samples, the PSP generated robust and accurate (>96%) scores and was significantly better (*p*<0.0001) than CgA. The assay protocol was consistent (r: 0.97) and reproducible (co-efficient of variation: 1.3–4.2%) across the two facilities.

**Conclusion:**

The PSP protocol for the NETest has been established and prospectively tested in clinical samples. It is highly reproducible, has similar metrics (CV, categorization by control or NET) to the standard PCR assay and generates clinically concordant (>96%) NETest results. Moreover, it functions significantly more accurately than CgA.

## Introduction

Critical issues in biomarker measurement are the requirement that the assay is highly reproducible, can be standardized and can be conducted in different laboratories with equal facility. This is particularly important when complex novel strategies are under development and assessment. Real-time PCR (qPCR) is the gold standard for nucleic acid quantification. Its strengths include sensitivity, a wide dynamic quantitative range, and the ability to reliably differentiate small differences in gene expression. The clinical utility of this strategy has been established and tests based on qPCR of selected genes e.g., MammaPrint or the Oncotype Dx family of assays, have become the standard for determining prognosis and specific therapeutic choice in oncology [1–6]. We developed and clinically validated a qPCR-based gene expression assay (TaqMan primer/probe sets from ThermoFisher) for diagnosing and managing gastroenteropancreatic neuroendocrine tumors (NETs) based on a 51-marker gene signature [7–10]. The assay—NETest—is undertaken in a CLIA-approved molecular diagnostics facility (Wren Laboratories, CLIA 07D2081388) and has been evaluated and the protocol and test accepted by the New York Department of Health (PFI: 9138). The assay is significantly more accurate than other biomarkers like chromogranin A (CgA) [11]. The NETest assay is based on isolation of circulating mRNA from whole blood, cDNA synthesis and then real-time PCR against 51 targeted genes that capture the biology of the neuroendocrine tumor cell [9]. The methodologies are standard but time-consuming since individual assays are run in 384-well plates.

Ready-to-use PCR plates, known as pre-spotted plates (PSP), consist of plastic supports for qPCR (e.g. 96-or 384-well plates) in which primers and probes from chosen assays are pre-dispensed and dried on plates. PSP were developed to facilitate multi-target analyses. Thus, a single reaction mixture per sample, containing all reagents except primers and probes, is prepared and loaded into the appropriate wells. This approach accelerates laboratory productivity and decreases the likelihood of error. An additional advantage is that it allows for a straightforward transfer of a qPCR-based assay to a collaborating laboratory facility that possesses molecular diagnostic skill-sets or the ability to automate the PCR-step. Successful examples range from the development of PSPs to screen for genetically modified organisms (GMOs) in food samples [12] to assays for multi-pathogen detection [13] to a 73 gene-based assay that evaluates the entire coding region of *BRCA1* and *BRCA2* [14]. These strategies were developed as time- and cost-effective methodologies that could produce reliable, reproducible results and are straight-forward to implement.

The exponential nature of PCR combined with small quantities of target or mispriming events, however, can result in substantial differences in the final result [15–19]. These considerations [20, 21] underscore the importance of stringent quality control during the validation of qPCR-based tools [22]. Wren Laboratories in collaboration with ThermoFisher constructed a specific NETest-PSP based on the 51-marker genes, and positive/negative controls established and required for CLIA-approved clinical testing. We evaluated the utility of these pre-loaded plates to run the NETest assay in a pilot and validation series of samples. We focused on the concordance of gene expression and output (algorithmic results) to stringently determine the utility of the PSPs. We then tested the assay in two different molecular diagnostic laboratories in separate countries as a further test of robustness of the assay. We also compared the assay to CgA in matched samples to verify its diagnostic utility. Finally, we examined whether the PSP could adequately replace the TaqMan-based assays for CLIA clinical samples by evaluating output in prospective, clinically requisitioned samples.

## Methods

### Study design

**Fig 1:**
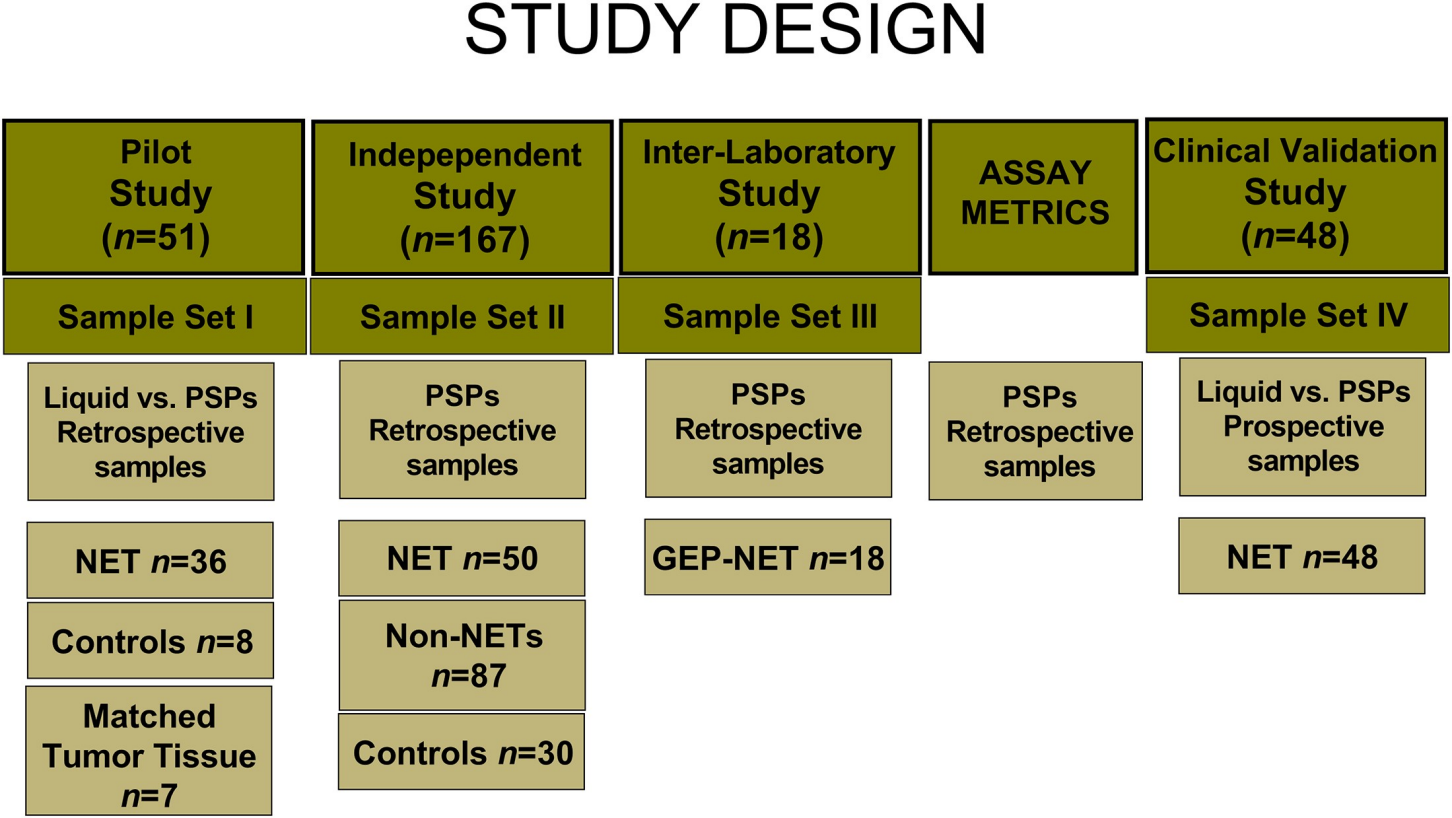
Consort diagram. The methodological approach taken to evaluate the circulating gene transcript assay in PSPs compared to standard liquid-based PCR. GEP-NET = gastroenteropancreatic NET. PSP = pre-spotted plate.

Several interlinking approaches were used to investigate the utility of PSPs. These, per STARD2015 criteria [23], are included (Fig 1). All samples were collected and analyzed according to an IRB specifically approved protocol (Yale University School of Medicine: HIC0805003870, approved 6/15/2016). Informed written consent was obtained from all study subjects. Blood and tumor tissue samples were collected prospectively between July 2017 and April 2019 from patients and non-affected family members (controls) of the patients attending the NET outpatient center or from patients undergoing clinical NETesting at Wren Laboratories (WIRB: 20150174, approved: 2/9/2015).

Briefly, 44 retrospectively collected samples (8 controls and 36 NETs) were evaluated using liquid approach and PSPs. The C_Q_ and normalized gene expression levels were compared to identify the utility of the plates to amplify target genes accurately. The output (NETest) was examined and compared to determine the reliability of the PSPs to generate an accurate score. Thereafter, a comparison was undertaken in matched blood and tumor tissue samples (*n* = 7) to verify that the PSP-NETest functioned as a liquid biopsy. One hundred and sixty-seven independently collected samples (30 controls, 87 non-NET controls and 50 NETs; samples deidentified before evaluation) were then tested on PSPs and the diagnostic utility of the test evaluated. Matched plasma samples were available for CgA analysis. Then, an inter-laboratory concordance evaluation was undertaken in two separate, accredited molecular diagnostic laboratories to investigate the robustness of the PSPs (*n* = 18 samples; deidentified). Laboratory metrics including inter- and intra-laboratory assessments were then undertaken. Finally, the PSP was prospectively examined in 48 consecutive clinical samples to establish a PSP protocol for a clinical laboratory. To further validate the clinical utility of the PSP protocol, we compared the NETest results with those of CgA undertaken at the same time-point.

### Clinical samples

Blood samples for the NETest were collected in 10.8mg K_2_EDTA tubes (BD Vacutainer Venous Blood Collection Tubes, BD Diagnostics). Aliquots of whole blood were stored at -80°C within 2 hrs of collection (samples immediately stored on ice/4°C after sampling) per standard molecular diagnostics protocols [24]. Blood samples for CgA were collected at the same time point in PPT plasma preparations tubes. Matched tumor tissue samples were available in seven cases. Tissue (*n* = 7) were collected at the time of surgery [25]. Samples were snap frozen in liquid nitrogen. Deidentified samples were sent to Wren Laboratories for RNA isolation and NETest PCR.

#### Sample Set I

Eight healthy controls were measured by standard and spotted plate PCR approaches respectively. Similarly, *n* = 36 NETs (lung: *n* = 4; pancreatic: *n* = 12; small bowel: *n* = 18; appendiceal *n* = 2) were assessed using both techniques. Matched tumor tissue was available from 7 GEP-NETs for analysis. This included 3 pancreatic NET and 4 small bowel NET.

#### Sample Set II

Thirty healthy controls, eighty seven non-NET controls (including GERD: *n* = 4; GIST: *n* = 6; IPMN: *n* = 16; pancreatic serous cystadenomas: *n* = 4; chronic pancreatitis: *n* = 2; incidentalomas: *n* = 5 [1x hyperparathyroidism, 2x nodular goiter, 2x unknown]; lung adenocarcinomas: *n* = 12; lung squamous cell carcinomas: *n* = 9; ampulla of Vater cancer: *n* = 1; pancreatic adenocarcinoma: *n* = 16; colon adenocarcinomas: *n* = 10, and 2 NECs [1x gastric G3 and 1x colon NEC]) and 50 NETs (lung: *n* = 5; gastric: *n* = 1; pancreatic: *n* = 20; small bowel: *n* = 22; rectal *n* = 2) were assessed using PSP. CgA was undertaken in matched plasma samples.

#### Sample Set III

Eighteen GEP-NETs (pancreatic: *n* = 7; small bowel: *n* = 11) were evaluated in two different laboratories (Wren Laboratories, CT, USA and Sarah Cannon Molecular Diagnostics [SCMD], HCA Healthcare UK, London). PSPs were used.

#### Sample Set IV

Clinical utility set: 48 prospectively collected NETs (lung: *n* = 5, gastric: *n* = 1; pancreas: *n* = 9, small bowel: *n* = 18; appendix: *n* = 9; colon: *n* = 2; rectal: n = 1; carcinoid of unknown primary: *n* = 3) were evaluated using both the standard (CLIA/liquid approach) and PSPs. CgA was also undertaken in matched plasma samples.

### Sample preparation and PCR

The studies were undertaken in a USA clinically certified laboratory (Wren Laboratories CL-0704, CLIA 07D2081388, New York Department of Health: PFI 9138, NETest: 55835). Sample Set III was also independently evaluated in the SCMD Facility, London, UK). The details of the PCR methodology, mathematical analysis and validation have been published in detail [26–28]. A 3-step protocol (RNA isolation with cDNA production and qPCR) was undertaken using a manual technique in the authors’ laboratory. Transcripts (mRNA) were isolated from 1ml EDTA-collected blood samples using the QIAamp RNA Blood Mini Kit (Qiagen). The RNA quality (A_260:280_ ratio) and quantity are measured using NanoDrop. The standard Qiagen isolation protocol (heme/gDNA contamination not detected) with no modifications was used. cDNA was produced from 50ul of eluted RNA using a High Capacity Reverse Transcription kit (Applied Biosystems: cDNA production 2000-2500ng/ul) and stored at -80°C.

#### Standard assay

Real-time PCR was performed (384-well plate) with the cDNA (200ng) and 16ul of reagents/well (Universal Master Mix II with UNG, Applied Biosystems, triplicate wells) (50°C 2 min, 95°C 10 min, then 95°C 15s, 60°C, 60s for 40 cycles). The majority of the 51 primers are exon spanning (82%, the remainder (18%) spanned one exon); all were <160bps (Table 1). For clinical testing a 7900HT qPCR machine was used. For all other testing, QuantStudio 7 Flex qPCR machines were used.

**Table 1:** Details of NETest genes (*n* = 51).

Target		Chromosome (Cytogenetic band)	UniGene ID
Symbol	Name		
AKAP8L	A kinase (PRKA) anchor protein 8-like	19p13.12	Hs.399800
ALG9	asparagine-linked glycosylation 9, alpha-1,2-mannosyltransferase homolog	11q23.1	Hs.503850
APLP2	amyloid beta (A4) precursor-like protein 2	11q24.3	Hs.370247
ARAF1	v-raf murine sarcoma 3611 viral oncogene homolog	Xp11.3	Hs.446641
ATP6V1H	ATPase, H+ transporting, lysosomal 50/57kDa, V1, Subunit H	8q11.23	Hs.491737
BNIP3L	BCL2/adenovirus E1B 19kDa interacting protein 3-like	8p21.2	Hs.131226
BRAF	v-raf murine sarcoma viral oncogene homolog B1	7q34	Hs.550061
C21ORF7 (MAP3K7 C-terminal like)	chromosome 21 open reading frame 7	21q21.3	Hs.222802
CD59	CD59 molecule, complement regulatory protein	11p13	Hs.278573
COMMD9	COMM domain containing 9	11p13	Hs.279836
CTGF	connective tissue growth factor	6q23.2	Hs.410037
ENPP4	ectonucleotide pyrophosphatase/ phosphodiesterase 4	6p21.1	Hs.643497
FAM131A	family with sequence similarity 131, member A, transcript variant 2	3q27.1	Hs.591307
FLJ10357 (ARHGEF40)	Rho guanine nucleotide exchange factor (GEF) 40 (ARHGEF40)	14q11.2	Hs.35125
FZD7	frizzled homolog 7 (Drosophila)	2q33.1	Hs.173859
GLT8D1	glycosyltransferase 8 domain containing 1, transcript variant 3	3p21.1	Hs.297304
HDAC9	histone deacetylase 9, transcript variant 6	7p21.1	Hs.196054
HSF2	heat shock transcription factor 2, transcript variant 1	6q22.31	Hs.158195
KRAS	v-Ki-ras2 Kirsten rat sarcoma viral oncogene homolog	12p12.1	Hs.505033
LEO1	Leo1, Paf1/RNA polymerase II complex component homolog (S. cerevisiae)	15q21.2	Hs.567662
MKi-67	antigen identified by monoclonal antibody Ki-67	10q26.2	Hs.689823
MORF4L2	mortality factor 4 like 2, transcript variant 1	Xq22.2	Hs.326387
NAP1L1	nucleosome assembly protein 1-like 1	12q21.2	Hs.524599
NOL3	nucleolar protein 3 (apoptosis repressor with CARD domain), transcript variant 3	16q22.1	Hs.513667
NUDT3	nudix (nucleoside diphosphate linked moiety X)-type motif 3	6p21.31	Hs.188882
OAZ2	ornithine decarboxylase antizyme 2	15q22.31	Hs.713816
PANK2	pantothenate kinase 2	20p13	Hs.516859
PHF21A	PHD finger protein 21A, transcript variant 1	11p11.2	Hs.502458
PKD1	polycystic kidney disease 1 (autosomal dominant), transcript variant 2	16p13.3	Hs.75813
PLD3	phospholipase D family, member 3, transcript variant 1	19q13.2	Hs.257008
PNMA2	paraneoplastic antigen MA2	8p21.2	Hs.591838
PQBP1	polyglutamine binding protein 1, transcript variant 2	Xp11.23	Hs.534384
RAF1	v-raf-1 murine leukemia viral oncogene homolog 1	3p25.2	Hs.159130
RNF41	ring finger protein 41, transcript variant 4	12q13.3	Hs.524502
RSF1	remodeling and spacing factor 1	11q14.1	Hs.420229
RTN2	reticulon 2, transcript variant 1	19q13.32	Hs.47517
SMARCD3	SWI/SNF related, matrix associated, actin dependent regulator of chromatin, subfamily d, member 3, transcript variant 3	7q36.1	Hs.647067
SPATA7	spermatogenesis associated 7, transcript variant 2	14q31.3	Hs.525518
SSTR1	somatostatin receptor 1	14q21.1	Hs.248160
SSTR3	somatostatin receptor 3	22q13.1	Hs.225995
SSTR4	somatostatin receptor 4	20p11.21	Hs.673846
SSTR5	somatostatin receptor 5, transcript variant 1	16p13.3	Hs.449840
TECPR2	tectonin beta-propeller repeat containing 2, transcript variant 2	14q32.31	Hs.195667
TPH1	tryptophan hydroxylase 1	11p15.1	Hs.591999
TRMT112	tRNA methyltransferase 11–2 homolog (S. cerevisiae)	11q13.1	Hs.333579
VMAT1 (SLC18A1)	solute carrier family 18 (vesicular monoamine), member 1	8p21.3	Hs.158322
VMAT2 (SLC18A2)	solute carrier family 18 (vesicular monoamine), member 2	10q25.3	Hs.596992
VPS13C	vacuolar protein sorting 13 homolog C (S. cerevisiae), transcript variant 2B	15q22.2	Hs.511668
WDFY3	WD repeat and FYVE domain containing 3	4q21.23	Hs.480116
ZFHX3	zinc finger homeobox 3, transcript variant B	16q22.3	Hs.598297
ZXDC	zinc finger C, transcript variant 2	3q21.3	Hs.440049
ZZZ3	zinc finger, ZZ-type containing 3	1p31.1	Hs.480506

#### PSP assay

Real-time PCR was performed with the cDNA (200ng) and 8ul of reagents/well (Universal Master Mix II with UNG, Applied Biosystems).

Raw C_Q_ values as well as normalized values (using *ALG9* and the ΔΔC_Q_ method [7, 29]) were calculated (Microsoft Excel). For generation of the NETest score, we used a MATLAB (R2011a) implementation of prediction approaches [7]. Target transcript levels are normalized and quantified versus a population control [26–28]. Final results are expressed as an activity index (NETest score) from 0–100% [26–28]. Normal score cut-off: 20%.

#### CgA ELISA

This was measured using NEOLISA Chromogranin A kit (Euro Diagnostica AB, Malmö, Sweden) at Wren Laboratories. The upper limit of normal was 108 ng/ml [28]. The laboratory is CLIA (07D2081388) and NYDOH certified (55834) for undertaking CgA clinical testing.

#### Statistics

In the pilot study (Sample Set I), intergroup analyses were undertaken using 2-tailed non-parametric tests (Mann-Whitney U test), Pearson correlations were used (for matched C_Q_ or gene expression samples: standard/PSP) and Predictive Modeling (PM) also undertaken. For PM, a historical NETest dataset [7] consisting of *n* = 158 Healthy Controls and *n* = 134 NETs was used to generate training and testing sets. Extreme Gradient Boosting (Xgboost), a supervised learning algorithm, was used to generate training and testing sets. Xgboost model was trained on a randomly generated stratified subset of samples consisting of 75% of all data, whilst the remaining 25% of data was used to assess model performance. Model hyper-parameter optimization was undertaken using Bayesian Optimization [30]. Briefly, 5-fold cross validation was performed to assess hyper-parameter effects and optimization was carried out on minimum child node weight, column subsamples per tree, maximum tree depth, subsample size, gamma, and alpha parameters. Learning rate was set to a constant 0.1 and training was undertaken over 3000 epochs with an early stopping at 50 epochs in cases where performance would not improve. Mean Absolute Error was used to assess model fitness. For tumor:blood pairs, normalized data were log-transformed and Pearson regression analysis undertaken as described [25].

In the Diagnostic group (Sample Set II), Mann-Whitney U-test and Area under the Receiver Operator Curve (AUROC) analysis was used to determine the diagnostic accuracy of the NETest [31–33]. Metrics calculated included sensitivity and specificity. To compare the utility of the PSP with CgA, McNemar’s test was undertaken in matched samples (NETs and non-NET controls) while diagnostic accuracies were directly compared (comparative ROC analysis) in the same groups.

In the inter-laboratory (Sample Set III) and the clinical validation (Sample Set IV) series, Mann-Whitney U-test and Pearson correlations were utilized. Inter- and intra-assay co-efficient of variation was undertaken per standard laboratory techniques [8]. Utility of the PSP to diagnose the NETest was directly compared to CgA using Fisher’s exact test.

Prism 7.0 for Windows (GraphPad Software, La Jolla California USA, www.graphpad.com) and MedCalc Statistical Software version 16.2.1 (MedCalc Software bvba, Ostend, Belgium; http://www.medcalc.org; 2017) were utilized. Statistical significance was defined at a *p* value < 0.05. Data are presented as mean±SD [median: Interquartile range].

## Results

### RNA yield and quality

We initially examined the yield and efficiency of RNA extraction. The RNA yield (*n* = 259 samples) was 12.1±18 [7.9:5.6–10.5]. The A_260:280_ ratio was 1.91±0.2 [1.86:1.81–2.05]. The protocol therefore generates adequate quality RNA for downstream applications.

### Concordance between standard plates and PSP for C_Q_ and normalized gene expression

**Fig 2:**
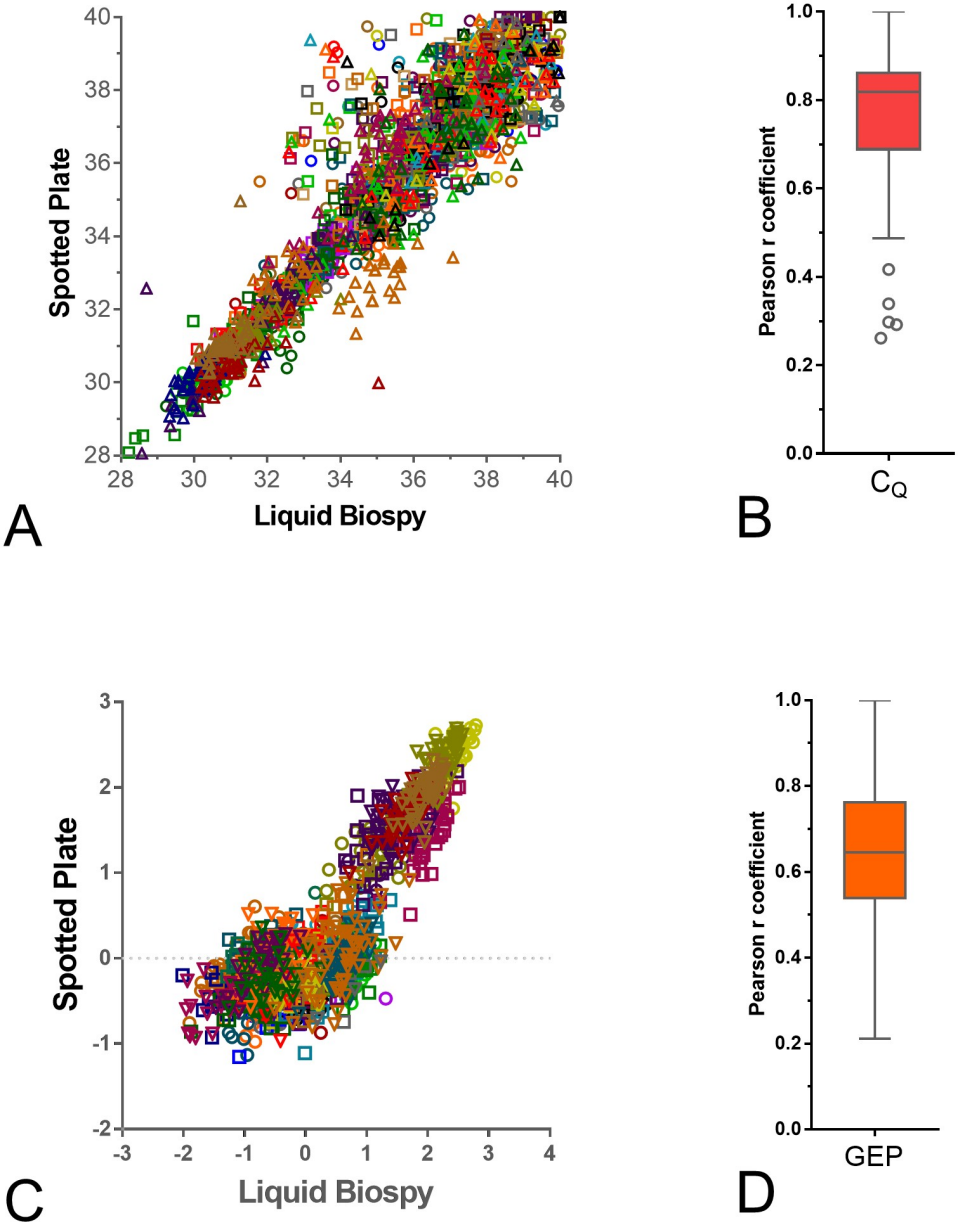
Concordance between C_Q_ and normalized gene expression levels in Sample Set I (*n* = 44). **2A/B**. C_Q_ levels. Scatter plot based on C_Q_ for each of the target genes (genes are individually colored) on spotted plates versus the standard approach (**2A**). The individual Pearson values ranged from 0.29–0.98. The Tukey box and whisker’s plot of the individual r-values (**2B**) identify the mean was 0.76 and median 0.82. **2C/D**. Gene expression. Scatter plot based on normalized gene expression for each of the target genes (individually colored) on spotted plates versus the standard liquid approach (**2C**). The individual Pearson values ranged from 0.21–0.98. The Tukey box and whisker’s plot of the individual r-values identify the mean was 0.65 and median 0.65 (**2D**).

We next compared C_Q_ values and normalized gene expression levels between the standard (liquid) and PSP approaches. We evaluated paired samples and calculated the Pearson values for the C_Q_ for each gene (Fig 2A and 2B) and the normalized gene expression (Fig 2C and 2D). These were highly and significant (*p*<0.001) concordant for the C_Q_ (0.76±0.18 [0.82:0.69–0.86]) (Fig 2A and 2B). Likewise, for normalized gene expression data, the Pearson r was highly concordant (*p* = 0.0048 –*p*<0.0001) (0.65±0.18 [0.65:0.54–0.76]) (Fig 2C and 2D).

### NETest (algorithm output) concordance between standard plates and PSP

**Fig 3:**
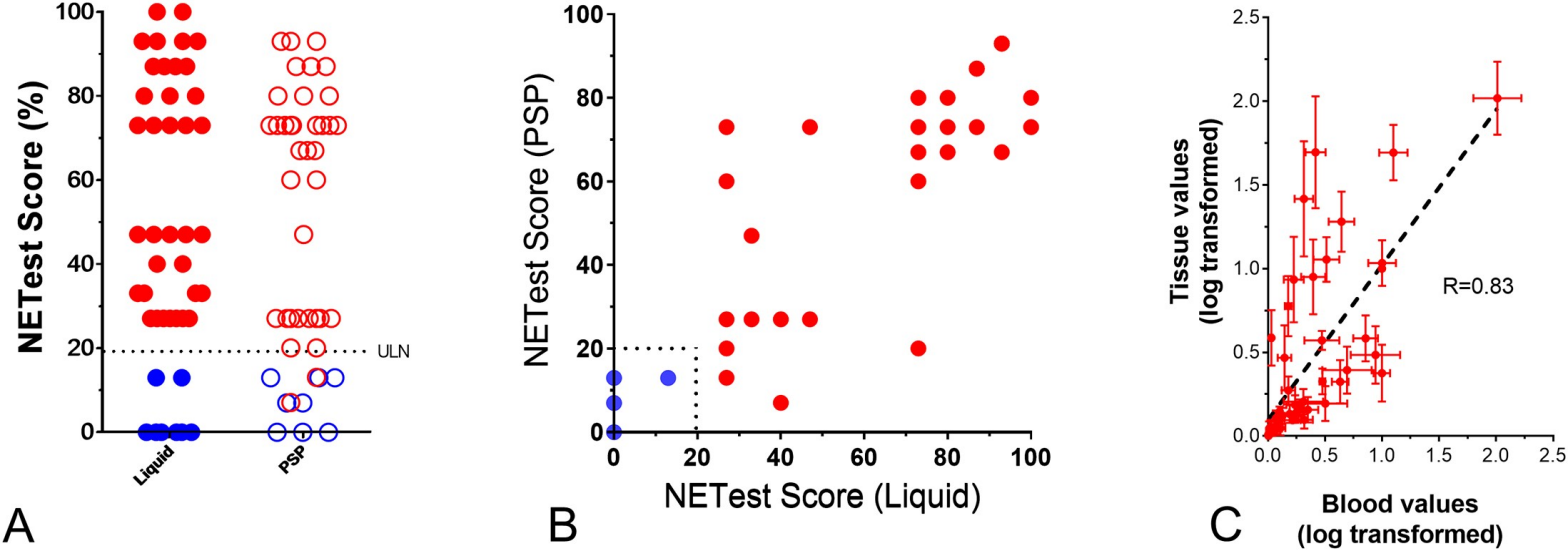
Relationship between NETest scores in the standard approach and PSPs in Sample Set I (*n* = 44). **3A**. The mean and median NETest scores were not significantly different (*p* = 0.674) between liquid (mean: 49.7; median: 47) and PSPs (mean: 48.5; median: 60). **3B**. The Pearson correlation for the NETest score was 0.873 (95%CI: 0.78–0.93), *p*<0.0001. Blue = controls (*n* = 8), red = NETs (*n* = 36). **3C**. Graph plot of the averaged log-transformed gene expression of matched blood-tissue pairs. Error bars indicate standard error of the mean. The dotted line represents the best linear fit line. Regression analysis identified R to range from 0.71–0.83 (*n* = 7, *p*<0.0001). ULN = upper limit of normal.

We next evaluated the utility of PSPs to deliver NETest scores equivalent to the standard approach. NETest levels were not significantly different (Wilcoxon matched pairs signed rank test: *p* = 0.674) between the standard liquid approach (48.6±33.6 [47:27–80]) and PSPs (48.5±31.5 [60:20–73]) (Fig 3A). The Pearson correlation r was 0.873 (*p*<0.0001) (Fig 3B).

### NETest (algorithm output) subgroup analysis

Thereafter, we used two methods to evaluate whether the NETest scores from the PSP differentiated between controls and NETs. In the first approach, we audited the forty-four samples from Sample Set I and, using the set cut-off threshold of 20 (on a scale of 0–100), evaluated how many samples fell into each category (normal: ≤20 or “NET” >20). The NETest scores for controls were completely concordant between the liquid approach and PSP—each identified 8/8 (100%) controls with scores less than 20 (Fig 3). All 36 NETs (100%) demonstrated NETest scores >20 using the standard approach. Thirty-four (94%) also had high NETest scores using the PSPs. The overall level of concordance between the standard approach and the PSP, therefore, was 42 of 44 (95.5%).

In the second method, we used Predictive Modeling (*xgboost*) to directly compare the NET vs. Control classification probabilities (liquid versus PSP) from Sample Set I against the initial set used to develop the original algorithm in 2013. This historical NETest dataset [7] consisted of *n* = 158 Healthy Controls and *n* = 134 NETs. Both standard PCR and spotted plate techniques differentiate NETs from Healthy Controls with comparable accuracies (average F1 Score of 0.98 and 0.95 for standard and spotted techniques respectively, Table 2).

**Table 2:** Concordance based on Predictive Modeling.

	Precision	Recall	F1 Score
Controls (standard)	1.00	0.89	0.94
NET (standard)	0.97	1.00	0.99
Controls (spotted)	1.00	0.86	0.92
NET (spotted)	0.92	1.00	0.96

### Concordance between gene expression in matched blood and tumor tissue

Finally, we evaluated the concordance between gene expression in matched tumor tissue and blood sample pairs (*n* = 7, including PNET: *n* = 3; small bowel NET: *n* = 4). The Pearson correlation (R) for the normalized gene expression in each of the tumor-blood paired groups was 0.71, and the Spearman correlation was 0.83 (Fig 3C). This was highly significant (*p*<0.0001).

### Validation of the PSP in an independent sample set

**Fig 4:**
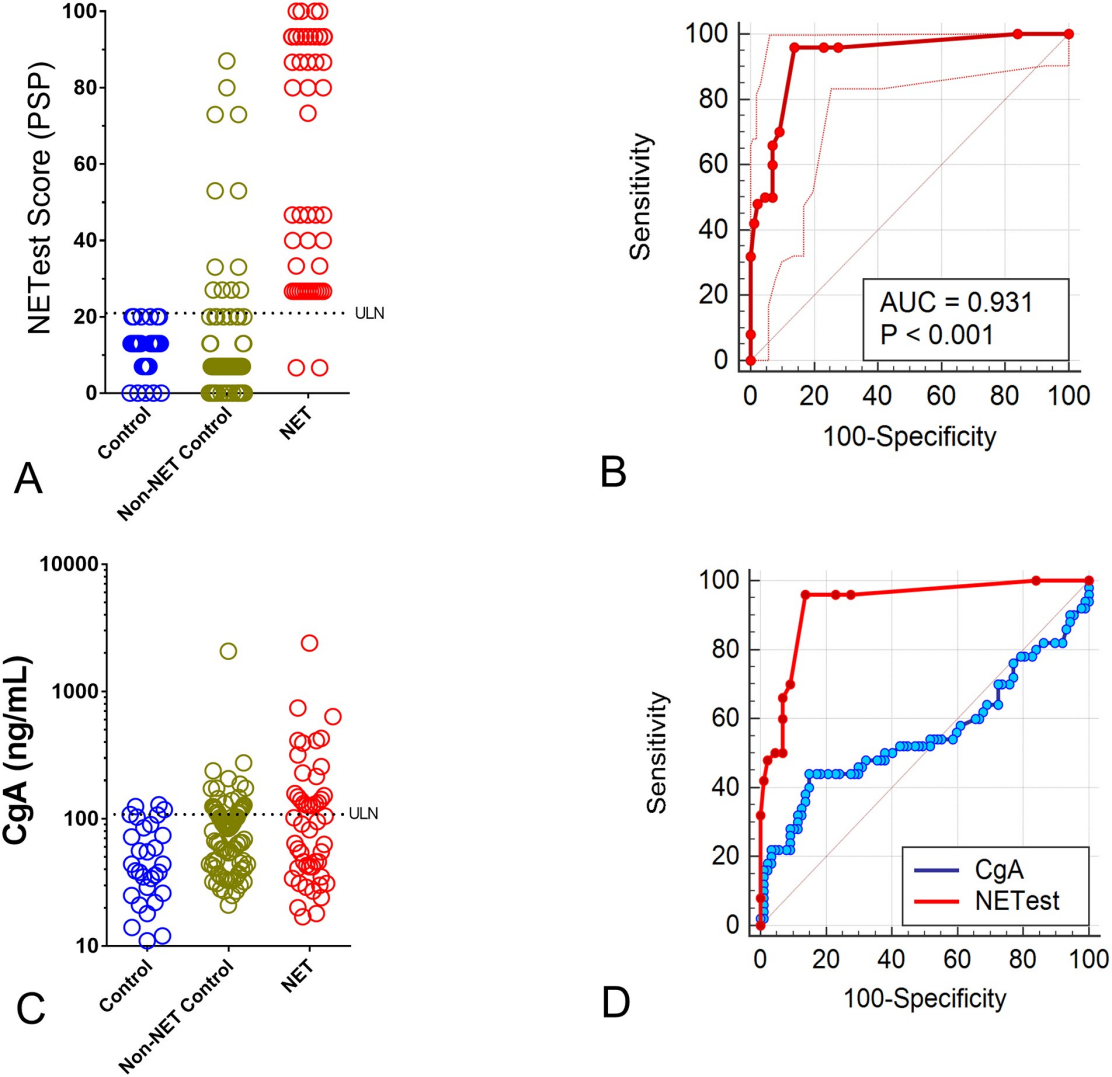
NETest scores and CgA levels in an independent validation cohort (Sample Set II, *n* = 167). **4A**. All 30 controls exhibited NETest scores ≤20. Twenty-eight (93%) of non-NET controls exhibited scores ≤20. Forty-eight (96%) of 50 NETs exhibited scores >20. **4B**. The AUROC for differentiating NETs from non-NET controls was 0.93±0.02 (95%CI: 0.88–0.97), *p*<0.0001. **4C**. Individual CgA measurements from the control, non-NET controls and NET samples using a standard CLIA ELISA approach. **4D**. AUROC comparison between the NETest and CgA for differentiating NETs from non-NET controls. The difference in AUC was 0.38±0.06 (95%CI: 0.26–0.49). The z-statistic was 6.44, *p*<0.0001. ULN = upper limit of normal.

A total of 167 real-life samples (Sample Set II) were then examined to independently validate the utility of the PSP to accurately diagnose a NET. Samples included 30 controls, 87 non-NET controls and 50 NETs. For the known controls, NETest scores were 11.2±6.5 [13:7–13]. For the non-NET controls, NETest scores were 13±18 [7:7–13]. For NETs, this was 60.9±31.4 [60:26.7–93] (Fig 4A). An area under the curve analysis identified an AUC of 0.97 for controls and 0.93 for non-NET controls. The Z-statistics were 27.1 and 19.9, while the Youden J indices were 0.93 and 0.82, respectively. The PSP exhibited a sensitivity of 96% and a specificity of 100% using a cut-off of 20 for controls. The sensitivity for non-NET controls was 96% with a specificity of 86% using the cut-off of 20. The overall concordance between distinguishing sample types was 153/167 (91.6%).

As a second evaluation, we compared the PSP-NETest results with the CgA results in matched samples. CgA levels were positive in three (10%) of controls, in 25 (29%) of non-NET controls and in 22 (44%) of NETs (Fig 4C). The AUROC for differentiating controls from NETs was 0.71; for non-NET controls, it was 0.56. A direct comparison between CgA and PSP-NETest identified the latter was significantly more accurate (123/137, 90%) than CgA alone (74/137, 54%) for differentiating NETs from non-NET controls. The McNemar’s test was highly significant (Chi^2^ = 20.0, *p* = <0.0001). A comparison of AUROCs confirmed that the PSP-NETest functioned significantly better than CgA for differentiating NETs from non-NET controls (difference in AUCs: 0.38, z-statistic: 6.44, *p*<0.0001; Fig 4D).

### Inter-laboratory concordance

To assess the robustness of the PSP-based NETest, two clinical laboratories (one from the USA and one from the UK) each tested 18 “real-life” matched blood samples (Sample Set III). Firstly, we examined the RNA quality metrics between the two laboratories. The RNA quantity was 5.5±2.6ng/ml (Laboratory 1 –Wren) versus 4.8±2 (Laboratory 2 –SCMD), *p* = 0.27. The RNA quality was 1.86±0.12 (Wren) versus 1.87±0.1 (SCMD), *p* = 0.89. The yield and efficiency therefore are consistent between the two laboratories.

**Fig 5:**
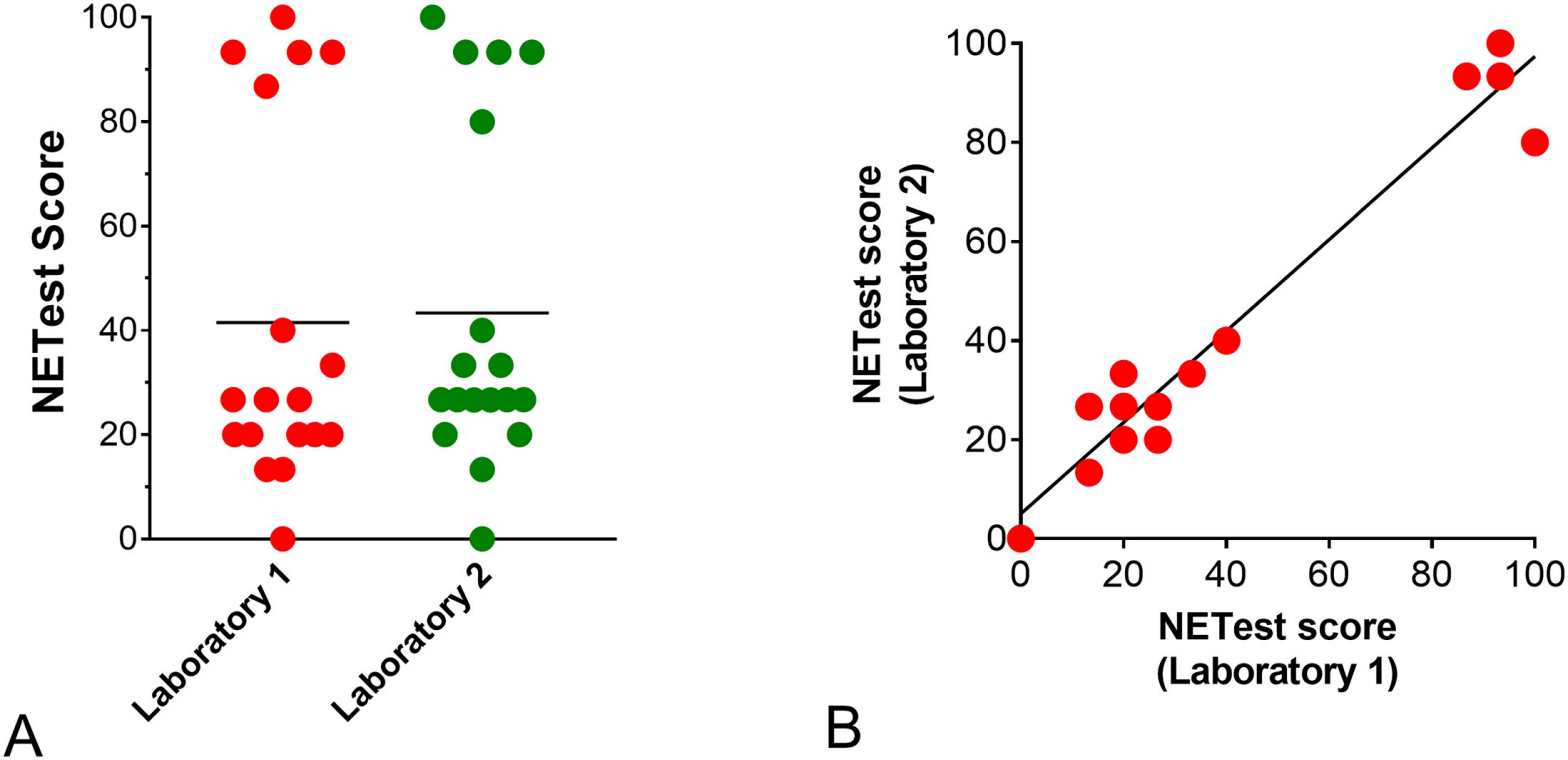
Robustness of PSP to attain a NETest result in Sample Set III (*n* = 18). **5A**. Individual scores from the 18 samples in each of the two laboratories. Scores were not significantly different. Horizontal line = mean value for each group. **5B**. Individual NETest correlations between the two laboratories (matched samples: *n* = 18). The correlation (Pearson r) was 0.967 (*p*<0.0001).

Secondly, we evaluated NETest scores from each of the two laboratories. These were 41.5±34.2 [26.7:25–83.3] (Wren) and 43.3±32.3 [26.7:20–88.4] (SCMD), respectively (Fig 5A). The Wilcoxon matched pairs signed rank test identified a *p* = 0.328. The Pearson correlation r was 0.967 (95%CI: 0.935–0.99), *p*<0.0001 (Fig 5B).

### Inter- and intra-laboratory reproducibility

**Fig 6:**
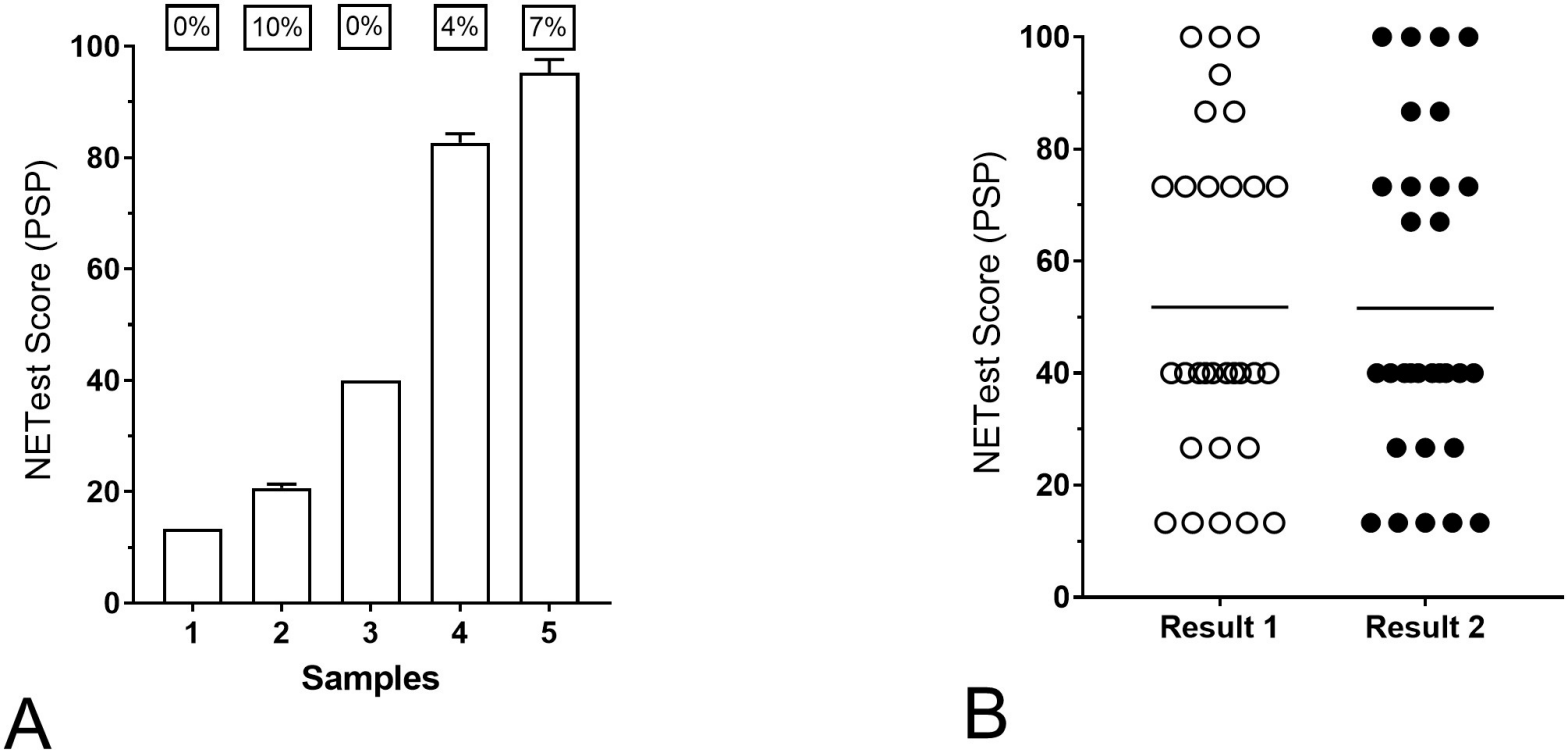
Reproducibility of the PSP assays. **6A**. Inter-assay reproducibility in five different samples. Each sample was repeated 5–10 times. The scores for each sample exhibited CV of 0–10%. The average CV was 4.2%. Bars are Mean+SD. **6B**. Intra-assay reproducibility. Thirty samples with NETest scores spanning the range of reported results were repeated (Result 1 vs. Result 2). The averaged CV for the 30 samples was 1.26%. Line = Mean; CV = co-efficient of variation.

For inter-assay reproducibility, multi-day (5–10 different days) reproducibility was examined. This was undertaken in 5 different clinical samples (Sample Set III). The metrics (co-efficient of variation) ranged from 0% to 10.4% (Fig 6A). The averaged CV was 4.24%. This value is similar to that defined for the liquid assay (5.9%) [8]. Between-samples/plate (intra-assay) variation was then evaluated in 30 clinical samples. The averaged co-efficient of variation was 1.26% (Fig 6B). This value is almost identical to that defined for the liquid assay (1.2%) [8].

### Clinical Testing Evaluation

**Fig 7:**
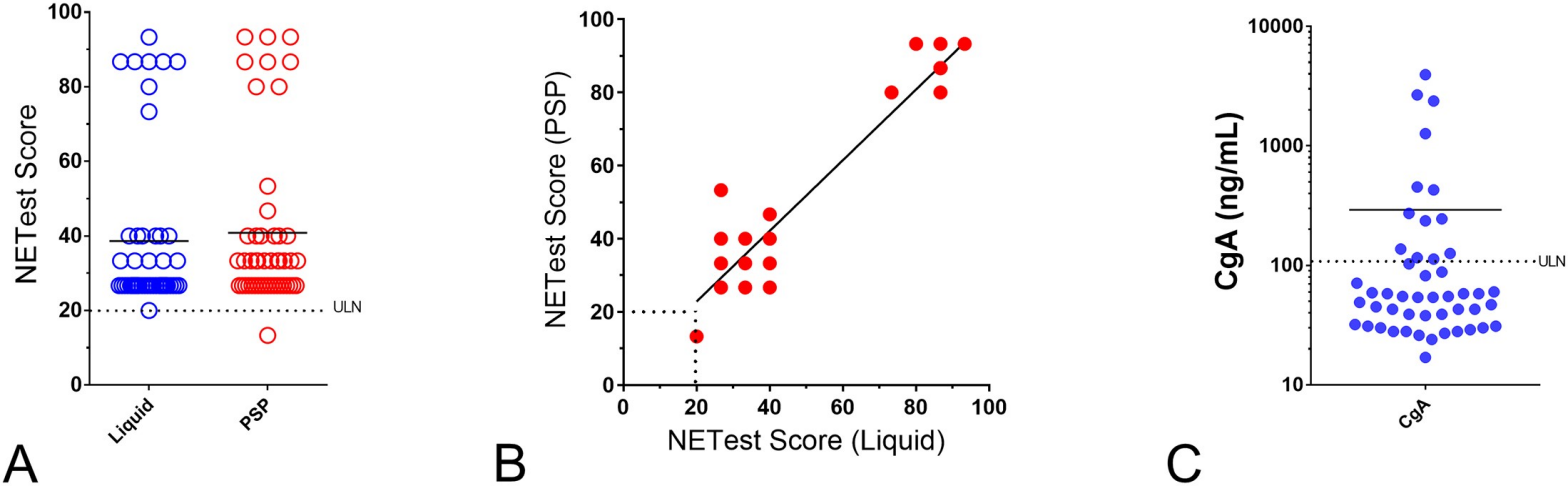
Robustness of PSP compared to CLIA-laboratory liquid sample results (Sample Set IV, *n* = 48). **7A**. Individual scores from the 48 consecutive samples using either standard CLIA approach (liquid) or on spotted plates (PSP). Scores were not significantly different. Horizontal line = mean value for each group. **7B**. Individual NETest correlations between scores on standard CLIA vs. PSPs (*n* = 48). The correlation (Pearson r) was 0.94 (*p*<0.0001). **7C**. Individual CgA measurements from the same 48 consecutive samples using a standard CLIA ELISA approach. Horizontal line = mean value for each group. ULN = upper limit of normal.

Finally, to assess the robustness of the PSP-based NETest, we directly compared NETest scores from liquid PCR and PSPs. Bloods were from 48 consecutive NET patients (Sample Set IV) ordered and undertaken under CLIA laboratory conditions and reported for clinical decision-making. NETest scores were 38.6±21.6 [26.7:26.7–40] (standard method, CLIA-reported) and 40.8±22.1 [33.3:26.7–40] (PSPs) (Fig 7A). The Wilcoxon matched pairs signed rank test identified a *p* = 0.675. The Pearson correlation r was 0.94 (95%CI: 0.899–0.968), *p*<0.0001 (Fig 7B). An examination of the variability across all the 51 marker genes identified this was significantly lower in the PSP-evaluated samples than for the standard liquid approach (median: 7.9 vs. 12.8, *p* = 0.0059).

An evaluation of outputs (NETest scores) identified 96% concordance between the standard approach and PSPs (Table 3). One of the samples categorized into the “low” score category (40) had an “intermediate” score (53) on the PSP, while a second sample with an intermediate score of 73 was called a high—score of 80 —on the PSP.

**Table 3:** Concordance between clinical sample outputs—Standard CLIA vs. PSP.

	CLIA	PSP	Concordance (%)
Normal score	1	1	100%
Low score (21–40)	39	38*	97%
Intermediate score (41–79)	1	0**	0%
High Score (≤80)	7	7	100%
	48	46	96%

*one sample was called intermediate on the PSP (NETest score = 53)

**the sample was called high on the PSP (NETest score = 80)

As a second evaluation, we compared the PSP-NETest results with CgA results (Fig 7C) in matched patient samples. More patients were NETest-positive (98%, 47/48) compared to CgA-positive (27%, 13/48). This was highly significant (Fisher’s exact test: *p*<0.0001).

## Discussion

The purpose of this manuscript was to standardize and establish the metrics of the PSP protocol with the aim of replacing the current liquid-based, CLIA-approved qPCR protocol. Our results confirm that a PSP platform can provide an acceptable replacement for the liquid-based NETest qPCR assay. The results demonstrated that the pre-spotted plates generated equivalent gene expression levels and outputs (scores) and were highly correlated with tumor tissue levels. Observations from the pilot study were independently validated and the diagnostic accuracy for the plates was confirmed to be >90%. The assay based on the PSPs was robust, standardized and easily transferrable to an independent molecular diagnostic facility. Furthermore, the PSPs generated clinically valid data concordant with liquid PCR under CLIA conditions. Finally, the PSPs, like the liquid-based NETest, was significantly more accurate than CgA as a diagnostic.

In our pilot study (*n* = 44), we examined the C_Q_ values between the standard (liquid) and PSP approaches and identified that these were highly concordant (r: 0.82) while the normalized gene expression was effectively reproduced on the plates (*p*<0.0001). An examination of the final output—gene expression conversion to a score—demonstrated that levels were highly correlated (r: 0.87) and not significantly different between the two approaches. An audit of these samples identified that the NETest scores for controls were completely concordant while almost all (34/36 or 94%) NETs studied had NETest scores >20 i.e., were called “tumor”. The overall level of concordance between scores was 95.5%. To independently confirm this, we compared the NETest scores against the original historical set used to develop the algorithm using Prediction Modeling. The average F1 Score for differentiating NETs from controls was 0.98 (standard) and 0.95 (PSP), confirming that the PSP technique was as accurate as the standard PCR approach. To validate that the PSP-NETest was an effective liquid biopsy, we directly compared matched tumor tissue and blood samples. The concordance was highly significant (R: 0.71–0.82, *p*<0.0001) and similar to what has been previously reported for the liquid-based assay [25, 34]. These data confirm that the PSP detects circulating tumor transcripts and functions as a *bona fide* liquid biopsy.

We next evaluated the utility of the PSP to differentiate between controls and NETs that were prospectively and independently collected. The NETest scores in the 30 known controls were <20 (mean: 11). In the 87 non-NET controls—including subjects with non-NET diseases that have been associated with clinical neuroendocrine features or a neuroendocrine phenotype (including, GERD, IPMN, pancreatic cysts, lung, pancreatic and colon adenocarcinoma), 75 were <20 (mean: 13). Two IPMN, one cystadenoma, 1 pancreatic adenocarcinoma, 2 colon cancers 2 lung adenocarcinomas and two squamous cell carcinoma were NETest-positive. Five of these NETest-positive samples also had elevated CgA levels. The positive NETest findings in IPMN and colon adenocarcinoma are consistent with observations from an earlier publication that identified minor overlap [35]. Similarly, positive NETest scores in lung neoplasia have recently been reported based upon the existence of neuroendocrine elements in lung cancer [36]. A histopathologically verified colon NEC and a G3 gastric NEC were both NETest-positive (score: 87 and 80, respectively) as would be predicted in tumors that have a neuroendocrine genotype and phenotype. The NETest genes have previously been independently confirmed as expressed in neuroendocrine carcinoma tumor tissues in a large NIH study of >10,000 tumors [37]. These authors (Chen *et al*.) also reported that 30–40% of histologically defined adenocarcinomas in the lung and gut expressed NET genes. This would be consistent with our observation that the two NEC patients would exhibit positive blood levels. Similarly, it is not unexpected that some lung and gut neoplasia will be NETest-positive. In the 50 NETs, the score was significantly (*p*<0.001) elevated (mean of 61) as compared to the non-NET conditions (mean of 13).

Forty-eight (96%) of the 50 were NETest-positive. Two patients, a rectal NET– 5 years post-surgery and a lung NET [typical carcinoid] 3 years post-surgery—both of whom had no evidence of disease, were therefore correctly identified. The AUROC analysis confirmed the test results from PSPs could be used as a diagnostic—the AUC was 0.97 versus controls and 0.93 versus non-NETs. Using the standard cut-off of 20 resulted in a diagnostic sensitivity of 96% and a specificity of 86–100% for the PSPs. The overall concordance between distinguishing sample types was 92%. In contrast, CgA measured in matched samples collected at the same time points was elevated in only 44% of NETs. The PSP-NETest, like the liquid-based PCR assay, was therefore confirmed to be significantly more accurate than a single analyte assay.

We next evaluated the robustness of the PSP-based NETest by comparing the process in two molecular diagnostics facilities, one in Branford, USA, the second in London, UK. Both facilities were provided with aliquoted blood samples (identical samples: *n* = 18). Each independently processed (from blood) and undertook cDNA synthesis and qPCR. NETest scores were not significantly different (mean: 42 vs. 43) and highly concordant (r: 0.97). This demonstrates that the protocol for diagnosing a NET using blood-gene expression is robust and that the PSP protocol can be adequately and appropriately transferred to other molecular technology familiar laboratories. The robustness of PSP approaches has previously been confirmed for a GMO PCR-screening assay. Nineteen different laboratories (throughout Europe) evaluated a 10-gene PSP to screen for GMOs in food samples [12]. The concordance ranged between 92–99%. Our inter-laboratory evaluation (97%) generated analogously accurate results.

We have previously evaluated the inter- and intra-assay metrics for the standard assay [8] demonstrating the inter-assay CV to be 5.9% and the intra-assay CV to be 1.2%. The assay on PSPs was as highly reproducible; the inter-assay CV was 4.24% and the intra-assay variation was 1.26%. In the UK laboratory, inter- and intra-assays were 2.2 and 1.2% respectively. These values demonstrate that the plates exhibit metrics that are at least as good as the standard assay [8]. Furthermore, an examination of the variability across all the 51 marker genes (Sample Set IV) identified that inconsistency was significantly lower in the PSP-evaluated samples than for the standard approach (median: 7.9 vs. 12.8, *p*<0.006). This further supports the consideration that pre-spotting PCR primers reduces the technical variability that may occur in liquid-based assays. This is especially likely when large numbers of genes (*n* = 51) are being evaluated.

Finally, to assess the robustness of the PSP-based NETest, we directly compared NETest scores from 48 consecutive NET patients and compared this to CgA. These were ordered and undertaken under CLIA laboratory conditions and reported for clinical decision-making to the same samples run on PSPs. Firstly, the PSP-NETest was identified to be significantly more effective than CgA as a diagnostic (*p*<0.0001). Secondly, NETest scores were concordant (mean: 39, CLIA-reported versus mean: 41, PSPs) and highly correlated (0.94), *p*<0.0001. Of note, the one clinical sample identified to have a normal NETest—a rectal NET two years after surgery and known to have no image or clinical evidence of disease—was appropriately identified by the PSP approach. We also examined the concordance based on categorizing the outputs into normal, low, intermediate and high categories of score—per CLIA and NYDOH requirements. The PSP results were highly concordant– 46/48 (96%)–with the standard approach. Two samples were misclassified. One sample exhibited a low score (CLIA/standard plate) of 40. On the PSP, the score was 53 which is a category change—from low to intermediate. The accuracy for “low” scores therefore is 97%. In this instance, the category change would be expected to result in a more careful follow-up of the patient [10]. The second sample had an intermediate score of 73 (CLIA/standard plate) whereas on the PSP, the score was 80 –a category change from intermediate to high. High scores are associated with a poorer prognosis [10]; in this instance, the physician would be alerted to the high score and the potential for an early relapse. PSP in two instances thus upgraded the risk category suggesting that this strategy might be effective in increasing the clinical vigilance required. A large study with far greater clinical patients would be required to assess this increase in efficacy.

In conclusion, the new spotted plate protocol for the NETest has been verified and prospectively tested in clinical samples. The spotted plate protocol is highly reproducible, has similar metrics (co-efficient of variation, concordance in output and overall categorization by control or NET) to the liquid assays, generates concordant NETest results and, like the liquid-based assay, functions significantly more accurately than CgA as a NET diagnostic. This approach is technically simpler than the standard liquid-based approach, uses less reagents and is more reproducible than standard protocols.

There is an increasing interest in different forms of liquid biopsies that can be utilized to guide patient care. Since such approaches are providing significant benefit and becoming increasingly integrated into clinical practice, we anticipate that the NETest will become a standard of care diagnostic for NET clinical management [38]. The NETest has demonstrable utility as a disease diagnostic. In addition, it can assess the effectiveness of surgery by the identification of residual or recurrent disease [27, 39, 40]. The NETest can also distinguish stable from progressive disease and monitor the effectiveness of medical treatment [10]. Given that our approach has been independently validated [34, 37], we anticipate that usage will become widespread. Clinical application and usage of this molecular assay will be facilitated by the PSP approach we describe. We anticipate that a PSP-NETest will provide both a significant cost savings for gene expression assays and allow for a broad introduction of the NETest assay into clinical laboratory practice.

## Supporting information

S1 Dataset All RNA information, sample types, NETest scores and CgA levels. (XLSX)
